# Explainable AI Model for Recognizing Financial Crisis Roots Based on Pigeon Optimization and Gradient Boosting Model

**DOI:** 10.1007/s44196-023-00222-9

**Published:** 2023-04-05

**Authors:** Mohamed Torky, Ibrahim Gad, Aboul Ella Hassanien

**Affiliations:** 1 Faculty of Artificial Intelligence, Egyptian Russian University, Badr City, Egypt; 2 Faculty of Science, Tanta University, Tanta, Egypt; 3 Faculty of Computer and AI, Cairo University, Cairo, Egypt; 4 Scientific Research Group in Egypt (SRGE), Cairo, Egypt

**Keywords:** Financial crisis, Explainable Artificial Intelligence (XAI), Machine learning, Pigeon Optimization

## Abstract

Utilizing Artificial Intelligence (AI) techniques to forecast, recognize, and classify financial crisis roots are important research challenges that have attracted the interest of researchers. Moreover, the Explainable Artificial Intelligence (XAI) concept enables AI techniques to interpret the results of processing and testing complex data patterns so that humans can find efficient ways to infer and interpret the logic behind classifying complex data patterns. This paper proposes a novel XAI model to automatically recognize financial crisis roots and interprets the features selection operation. Using a benchmark dataset, the proposed XAI model utilized the pigeon optimizer to optimize the feature selection operation, and then the Gradient Boosting classifier is utilized to recognize financial crisis roots based on the obtained reduct of the most important features. The practical results showed that the *short-term interest rates feature* is the most important feature by which financial crisis roots can be detected. Moreover, the classification results showed that the built-in Gradient Boosting classifier in the Pigeon Inspired Optimizer (PIO) algorithm achieved training and testing accuracy of 99% and 96.7%, respectively, in recognizing financial crisis roots, which is an efficient and better performance compared to the random forest classifier.

## Introduction

In the new global economy, financial crisis recognition and prediction have become a huge challenge for avoiding the bankruptcy of financial institutions and companies. Therefore, the early detection of financial crisis warning alerts is a great concern for financial policymakers to reduce the likelihood of bank failure and avoid rising risks [1]. Although the financial crisis has various dimensions and depends on plenty of financial and political variables, it can result in one or more of the following patterns [2], (1) currency crisis is a situation in which the national central bank has not to have enough foreign currency to maintain the country's fixed exchange rate. This leads to a rapid and sharp decline in the value of a country's currency, which harms the economy of that country, (2) sovereign crisis is a situation in which the country cannot pay its government debt or rescue indebted banks under its national financial control without the dependence of financial third parties like the International Monetary Fund (IMF) or the European Central Bank (ECB), (3) banking crisis is a situation in which many banks in a country are suffering from serious liquidity or financial solvency problems at the same time. The bankruptcy of one bank or a set of banks propagates this failure to other banks in the financial system. Therefore, the amount of non-performing loans is rapidly increasing, most of the banking system's capital is rapidly depleting, and (4) asset price correction (or market correction) crisis is a situation in which asset prices or financial market are decreased by 10–20% or greater. This correction can continue from days to months or even longer. Although market correction may be a damaging crisis in the short period, it can be positive for adapting overvalued asset prices and granting buying chances.

The causes of the financial crisis are diverse, forked, and unpredicted. Some of them are due to false execution of financial procedures such as excessive leverage, liquidity mismatches (lending long, lending short), false assessment of taxes and subsidies, and increased borrowing by banks and investors [3]. Moreover, financial crises can occur due to sudden global events as occurred with the COVID-19 pandemic [4]. Therefore, there is no standard definition of the financial crisis; however, it can be described as a sharp and sudden disturbance in some economic balances, followed by the collapse of several financial institutions, and its effects on others. The International Monetary Fund (IMF) revealed, in 2022, the global economic outlook and said that it will be bleaker than expected last year. While he referred to the significant deterioration in purchasing managers' surveys in recent months, as they indicated the weakness of most of the G-20 economies. Moreover, IMF wrote in a blog prepared for the G20 leaders' summit in Indonesia that the latest indicators "confirm that the outlook is bleaker", especially in Europe. He added that recent purchasing managers' indices measuring manufacturing and services activity show weakness in most of the G20 economies, with economic activity expected to contract amid high inflation. Moreover, it is expected that the exacerbation of the energy crisis in Europe will inflict severe damage on growth and will raise inflation, which, if it continues to rise, may lead to greater increases in interest rate policy and further tightening of global financial conditions. In addition, IMF explained that this, in turn, poses increased sovereign debt crisis risks for weak economies. He also added that the multiplicity of dangerous weather events will harm growth around the world [2, 3].

There has been an increasing interest in using computational intelligence and Artificial Intelligence (AI) in financial applications such as asset pricing and derivatives, stock market predictions, algorithmic trading, and credit risk assessment [5, 6]. Specifically, machine learning and deep learning technologies started getting many researchers' attention to develop a smarter model for recognizing, predicting, and classifying financial crisis roots [7] than classical methods that depend on complex calculations as in statistical and operations research approaches [8]. Compared to classical approaches, AI-based methods have two major strengths in recognizing financial crisis roots. The AI-based method can process and learn from unbalanced and random data. This means that the statistical distribution of the financial data is not necessary for AI-based methods to recognize financial crisis roots. Another strength of AI-based methods is that they can be categorized as nonlinear approaches. This advantage makes AI-based methods to be more accurate when testing complex financial data patterns [1]. In addition, a novel concept has emerged. It is associated with AI that enables machine learning and deep learning models to interpret the results of processing and testing complex data patterns so that humans can not only find solutions to complex data patterns problems but also understand and interpret the logic of the obtained solutions. This new concept is called Explainable AI (XAI), or Interpretable AI [9–11]. XAI can be a magic solution to the financial transparency challenge in financial services. The recent AI models can enhance many financial services' accuracy, speed, and effectiveness, such as predicting liquidity balances, assigning credit scores, and optimizing investment portfolios. However, the stakeholders (e.g., banks, investors, employees, customers, etc.) must understand the logic and reasoning behind applying these AI models to avoid financial bias. Therefore, XAI can answer this challenge [12]. Although XAI standards are still being built and the market for this technology is developing, current trends imply that Explainable AI will be a significant need for incoming economic AI applications. According to the Gartner website, by 2025, 30% of large companies and government contracts for purchasing AI applications will require explainable and ethical AI [13].

Moreover, several startup leaders in Explainable AI started to raise their investment in XAI dependence. For instance, Fiddler Labs recently allocated $14 million to assist in interpreting disparities in machine learning approaches and assure compliance with industry laws based on explainable AI techniques [14]. These reports indicate that financial organizations will have to provide evidence for all financial decisions to regulators and their customers, particularly if the results of an AI model are inaccurate. Consequently, XAI in institutions' financial work will prepare the way for the future of the global economy.

This paper proposes an Explainable Artificial Intelligence model to recognize the financial crisis roots in financial datasets. The main contribution of this study is:

- A novel XAI model is proposed to recognize financial crisis roots based on Pigeon Optimization (PIO) and Gradient Boosting classifier.
- The PIO algorithm is applied to optimize the financial crisis features selection step to obtain the best reduct of features that have the most impact on financial crisis recognition
- Two learning models, Gradient Boosting (GB), and (Random Forest) are applied and compared with the proposed XAI model to classify and recognize financial crisis roots
- The proposed model has been tested and validated in a benchmark financial crisis dataset. The experimental results of the proposed XAI model achieved training and testing accuracy of 99% and 96.7%, respectively, in recognizing financial crisis roots.

The remaining sections of this paper are organized as follows. Section 2 outlines the related work. Section 3 presents background about two major algorithms. Section 4 discusses the proposed XAI model for recognizing financial crisis roots. Section 5 presents the experimental results. Section 6 discusses the obtained findings. Section 7 concludes this work.

## Related Work

The literature on Artificial Intelligence to predict financial crisis roots has highlighted several techniques and methodologies [1, 15–17]. Over the past decade, most research has emphasized machine learning techniques to solve financial crisis forecasting problems.

Aristeidis et al. [18] proposed a machine learning-based monitoring system model called "Early Warning Systems” (EWS) to forecast financial crises based on the structure and analysis of financial networks. The proposed models achieved an accuracy of 98% in predicting the financial crisis.

Sotirios et al. [19] studied a stock market crisis prediction based on statistical and deep learning approaches. The authors investigated the efficiency of six models, Support Vector Machines (SVM), Extreme Gradient Boosting, Classification Trees, Random Forests, Neural Networks, and Deep Neural Networks, to predict stock market crisis cases. The experimental results proved that there is clear evidence of cross-contagion and interdependence impacts among stock, bond, and currency markets. Moreover, the results proved that the Deep Neural Networks approach optimizes sorting accuracy while providing a powerful method to build a systemic, efficient, and risk-sensitive early warning routine better than the currently established ones.

An additional stock crisis prediction model has been proposed and applied in the Indian stock market [20]. The authors applied a novel prediction model for stock crisis based on a practical comparison between Deep Neural Network (DNN) regression method and Extreme Gradient Boosting (XGBoost) using a proposed Hybrid Feature Selection (HFS) technique. The experimental results proved the superiority of XGBoost performance regarding Mean Absolute Error (MAE), Mean Squared Error (MSE), and Root Mean Square Error (RMSE) in predicting Indian stock market crises.

EeroTölö in [21] investigated the efficiency of selected traditional neural network models against the Gated Recurrent Unit (RNN-GRU) and Long-Short Term Memory (RNN-LSTM) for predicting financial crisis roots. The selected models have been validated on the Jórda–Schularick–Taylor dataset, which involves the crisis dates and annual macroeconomic series of 17 countries over the period 1870−2016. The experimental results proved that (RNN-LSTM) and (RNN-GRU) are efficient models for forecasting financial crises compared to the benchmark logistic model and basic neural nets.

Godwin et al. [22] investigated the efficiency of using the Light Gradient Boosting Machine (LightGBM) model for predicting credit risks and crises. The experimental results clarified that LightGBM prediction performance outperformed other models such as SVM and logistic regression.

Zukerman Rustam and Glori Stephani Saragih [23] investigated the utilization of the Random Forest (RF) model for predicting the financial crisis of Turkish banks. The simulation results clarified that RF achieved a competitive result in forecasting bank crises compared with other machine learning algorithms in the literature.

On the other hand, forecasting financial intermediaries' financial crises is very important. Stacey and Vlado [24] investigated a novel solution to this problem using the Convolutional Neural Networks model. The experimental results clarified classification accuracy, 88.24% to financial intermediaries as distressed or not distressed.

Financial crises lead to bank failures and insolvency. Youness et al. [25] introduced an empirical study using a Deep Neural Network approach for bank failure forecasting. The proposed model has been validated on a dataset collected over 14 years from 2004 to 2018. It consists of 1100 FDIC-insured US commercial banks. The experimental results clarified the outperformance of the proposed Deep Neural Network model compared with other traditional machine learning models and statistical techniques in the Matthews Correlation Coefficient and F1-score.

Hong Hanh Le and Jean-Laurent Vivian [26] conducted a comparison study between classical statistical models (i.e., Logistic Regression and Discriminant Analysis) and machine learning models (Support Vector Machines, Artificial Neural Network, and k-Nearest Neighbors) in forecasting banks' insolvency cases. The authors verified the comparison study. A dataset consists of 3000 US banks. The practical result proved the efficiency of Artificial Neural Networks and k-nearest neighbor methods in predicting bank failure compared to the SVM and statistical approaches.

Nurul Alam et al. [27] introduced an efficient deep learning algorithm using a panel data structure to forecast corporate bankruptcy. The authors compared the proposed deep learning model with the known discrete hazard model (it has been widely used in panel data applications through finance literature). They validated the proposed model's performance on a dataset of 641,667 firm-month observations of North American listed companies between 2001 and 2018. The practical results clarified the outperformance of the proposed deep learning model with a prediction accuracy of 93.71%, compared with the discrete hazard model, which achieved low accuracy of 86.95%.

Al-Haschimi et al. [28] proposed a machine learning technique for measuring the overall financial risk in China based on textual data. Latent Dirichlet Allocation (LDA) algorithm has been utilized to identify major episodes of financial risks in China based on the textual data from many newspaper articles. A Structural Vector Autoregressive (SVAR) model, is then used to quantify the impact of rising financial risk on the Chinese and the global economy.

Bitetto et al. [29] used multiple versions of the principal component analysis (PCA) approach to develop a novel index that can be used to measure the soundness of a country’s financial system around the world based on machine learning. Testing results have shown that the proposed index makes robust forecasting about the soundness of financial systems for 119 countries, with very good results.

Medianovskyi et al. [30] proposed Explainable Artificial Intelligence (XAI) model for predicting financial disasters in Small and Medium Enterprises (SMEs). The authors applied Gradient Boosting (XGBoost and Catboost), Random Forests, Logistic Regression, and Artificial Neural Networks as regression models, then used Shapley’s Additive Explanations (SHAP) framework for modeling result interpretation and validation of financial crisis prediction. The experimental results have shown that the most accurate way to evaluate financial crisis for SME enterprises is by utilizing the Catboost modeling technique. Moreover, it was specified that SMEs that are younger than 10 years old and have a cash ratio below 20% are significantly more likely to face a financial crisis. The result also finds that ‘hazardous’ feature combinations have a higher (or lower) impact on financial crisis occurrence.

Swarm optimization approaches such as Ant Colony Optimization (ACO) [31], Particle Swarm Optimization [32], Grey Wolf Optimization (IGWO), and Fuzzy Neural Classifier (FNC) [33] can be used also to develop smart systems able to accurately predict the financial crisis and bankruptcy. Although many appreciated efforts have been paid to solve financial crisis prediction issues, few works have used the concept of Explainable Artificial Intelligence (XAI) in predicting financial crises. Moreover, more enhancement in prediction accuracy is required while applying novel machine learning models. These two challenges are what we investigate in this study.

## Background

This section discusses two major techniques in which the proposed system is built, the Pigeon Swarm Optimizer, and the SHAP algorithm. The next two subsections discuss the purpose, and methodology of each algorithm.

### Pigeon Swarm Optimizer

In 2014, Duan developed a new swarm intelligence optimization technique known as Pigeon Inspired Optimization (PIO). Numerous optimization issues have been effectively solved using population-based swarm intelligence systems. All bio-inspired optimization algorithms attempt to imitate natural ecosystem systems, which have substantially enhanced the practicality of modern optimization approaches and provided practical solutions to those complex combinatorial optimization problems [34]. In this paper, we will adapt the new PIO algorithm proposed by Duan and Qiao [35] that mimics the real navigation process of pigeons. The algorithm contains two main operators as defined as follows.

1. Map and compass operator: Pigeons form a map in their brains when they sense the field of the Earth using magnetic reception and look at the sun's height as a compass to adjust direction as they depend less on magnetic particles and the sun when they fly to their final destination.
2. Landmark operator: Pigeons rely on their nearby landmarks when they fly near their destination as they fly directly to the destination. If they are familiar with the landmark, they will fly directly to the destination while following the pigeon that knows the destination if they are far from the destination and unfamiliar. The number of pigeons was halved in each iteration, the first half of the population was categorized as the current population, the other half of the poor were abandoned, and a new population center was identified.

**Fig. 1:**
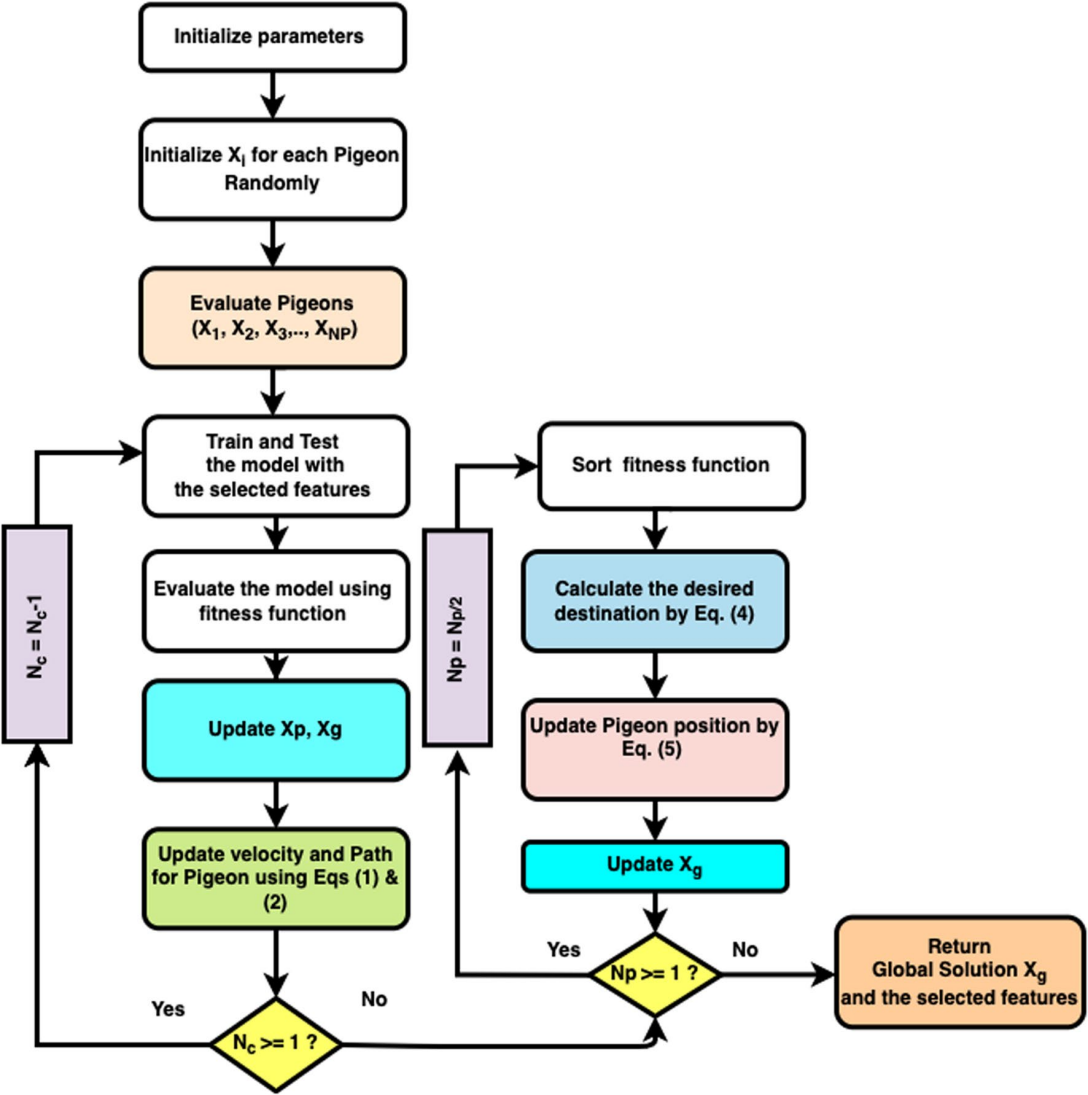
The flowchart of the PIO algorithm

The main steps of PIO are described in Fig. 1. The first step is used to initialize the parameters of the PIO algorithm such as the initial random set of pigeons, compass operator R, the solution dimension space, number of iterations, and size of the population. Next, randomly initialize the position ${\mathrm{X}}_{\mathrm{i}}(\mathrm{t})$ for each pigeon. At the $t$ iteration, the machine learning model is trained and tested using the selected features. Moreover, the model is evaluated using the fitness function. Next, the following changes are implemented to the position ${\mathrm{X}}_{\mathrm{i}}(\mathrm{t})$ and velocity ${\mathrm{V}}_{\mathrm{i}}\left(\mathrm{t}\right)$ of each pigeon in the group as depicted in Eqs. 1 and 2:

1 $${\mathrm{X}}_{\mathrm{i}}(\mathrm{t})= {\mathrm{X}}_{\mathrm{i}}\left(\mathrm{t}-1\right)+{\mathrm{V}}_{\mathrm{i}}(\mathrm{t})$$

2 $${\mathrm{V}}_{\mathrm{i}}\left(\mathrm{t}\right)={\mathrm{V}}_{\mathrm{i}}\left(\mathrm{t}-1\right) {\mathrm{e}}^{-\mathrm{R}(\mathrm{t})}\mathrm{ rand }\left({\mathrm{X}}_{\mathrm{g}}- {\mathrm{X}}_{\mathrm{i}}\left(\mathrm{t}-1\right)\right)$$

where $R$ is the map factor and is the random number between 0 and 1, $\mathrm{rand}()$ represents the uniform random number, and ${\mathrm{X}}_{\mathrm{g}}$ indicates that the optimal position of the whole group of pigeons at t time is attained by selecting the individuals with the maximum fitness value after comparing the location of the entire flock, which is analogous to the direction indicated by the compass. Following a predetermined number of iterations, the ${\mathrm{X}}_{\mathrm{i}}$ is then transferred to the landmark operator to continue the iteration process.

In the landmark operator, the pigeons are arranged based on their fitness value. The ${N}_{landmark}\left(t\right)$ represents the pigeon’s number at iteration t as shown in Eq. 3. During each iteration, half of the pigeons are discarded since they are located too far from the destination, the center of the left pigeons and the pigeons that are closer to the destination can fly there faster. ${X}_{c}\left(t\right)$ represents the center location of the pigeon and is defined as in Eq. 4.

3 $${N}_{landmark}\left(t\right)=\frac{{N}_{landmark}\left(t-1\right)}{2}$$

4 $${\mathrm{X}}_{\mathrm{c}}\left(\mathrm{t}\right)=\frac{\sum {\mathrm{X}}_{\mathrm{i}}\left(\mathrm{t}\right)*\mathrm{fitness}({\mathrm{X}}_{\mathrm{i}}(\mathrm{t}))}{{\mathrm{N}}_{\mathrm{landmark}}\left(\mathrm{t}\right)*\mathrm{ fitness}({\mathrm{X}}_{\mathrm{i}}(\mathrm{t})}$$

where $fitness\left({X}_{i}\left(t\right)\right)$ is the fitness value of each pigeon, $fitness\left({X}_{i}\left(t\right)\right)$ is the objective function value corresponding to the given pigeon's position, and ${N}_{landmark}\left(t\right)$ is the current reduced population. The new position is defined as in Eq. 5. Finally, pigeons update their positions using the best center position of each iteration. Through these two parts of updates, pigeons will soon find the global best position in history.

5 $${X}_{i}\left(t+1\right)={X}_{i}\left(t\right)+rand\left({X}_{c}\left(t+1\right)-{X}_{i}\left(t\right)\right)$$

6 $${V}_{i}\left(t+1\right)={V}_{i}\left(t\right) {e}^{-R\left(t\right)} rand\left({X}_{c}\left(t\right)-{X}_{i}\left(t\right)\right)$$

### Shapley Additive Explanations (SHAP) Algorithm

The National Institute of Standards (NIST) has proposed four principles to define Explainable Artificial Intelligence (XAI): (1) an AI system should supply proof, or reasoning for each of its outputs; (2) the explanations produced by an AI system should be comprehensible to its users; (3) the explanations should accurately depict the process utilized by the AI system to obtain the output; (4) the AI system should only function within the parameters for which it was designed, and should not provide an output when its confidence in the result is insufficient.

The SHAP values provide a high level of interpretability for a model. The SHAP is a Python-based library developed by Lundberg and Lee [36] to explain an ML model's results by evaluating the impact of each feature on the model's output. This technique assesses the relative importance of a particular local feature using the Shapley values [37, 38] from game theory. The SHAP values provide two great advantages (1) global interpretability: the SHAP values can show how much each predictor contributes to the target variable, either positively or negatively. This is similar to the plot of variable significance, but it can illustrate the positive or negative link between each variable and the target and (2) local interpretability; the SHAP values for each observation are unique. However, traditional variable significance algorithms do not provide each example's findings. Local interpretability can be used to compare the effects of different factors [39, 40]. The Shapley values (${\mathrm{\varnothing }}_{\mathrm{i}}$) for a particular feature i can be calculated by taking the average of the marginal contribution of feature i computed for all subsets S of N, excluding feature i [41], as shown in Eq. 7.

7 $${\mathrm{\varnothing }}_{\mathrm{i}}=\frac{1}{\mathrm{n}!}\sum_{\mathrm{S }\subseteq \mathrm{N}-\{\mathrm{i}\}}\left|\mathrm{S}\right|!\left(\mathrm{n}-1-\left|\mathrm{S}\right|\right) ![\mathrm{f}(\mathrm{S}\cup \left\{\mathrm{i}\right\})-\mathrm{f}(\mathrm{s})]$$

where $S$ is a subset of features, f is the prediction model, $N$ is the set of all features, and $n$ is the total number of features. For a single observation, $\mathrm{x}$, a linear function $\mathrm{g}$ is used to explain the output of the prediction model and can be calculated from Eq. 8.

8 $$\mathrm{f}\left(\mathrm{x}\right)=\mathrm{g}\left({\mathrm{x}}^{*}\right)={\mathrm{\varnothing }}_{0}+\sum_{\mathrm{i}=1}^{\mathrm{M}}{\mathrm{\varnothing }}_{\mathrm{i}}{\mathrm{x}}_{\mathrm{i}}^{*}$$

where $\mathrm{x}$ is the instance being explained, ${\mathrm{x}}^{*}$ represents the input being simplified, $\mathrm{M}$ is the number of simplified input attributes, ${\varnothing }_{0}$ is the initial value when none of the inputs are present.

## The Proposed Financial Crisis Roots Recognition System

**Fig. 2:**
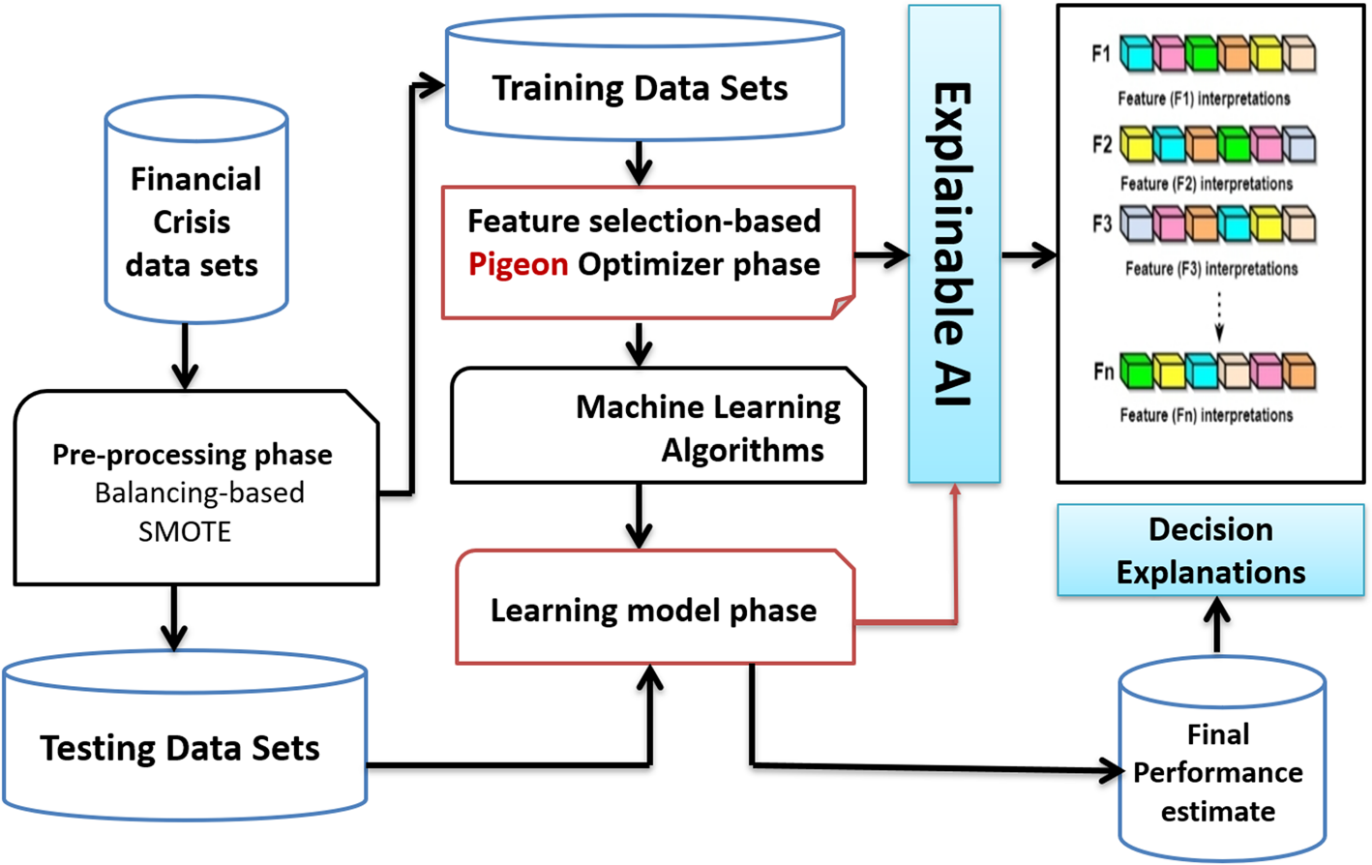
The general architecture of the proposed financial crisis roots recognition system

The proposed Explainable AI system comprises three main phases: (1) pre-processing phase, (2) feature selection based on the Pigeon Swarm Optimization algorithm, and XAI-based interpretation phase, and (3) financial crisis roots recognition based on machine learning and XAI phase. Figure 2 depicts the proposed system's overall architecture. In the next subsections, each of the three phases, including the actions required and the characteristics and features of each phase are explained in more detail.

### The Pre-processing Phase

In machine learning-based problems, one of the essential stages in developing classification algorithms in data science is balancing the data. Without balancing train data, classification models will generally perform ineffectively. One of the common quick, easy, and effective ways to accomplish this important task is SMOTE, which stands for "Synthetic Minority Oversampling Technique." It creates new data based on the implications of old data. Instead of removing or copying existing data, new rows will be produced and labeled according to the original data. Table 1 shows the number of samples in both two classes before and after applying the SMOTE approach.

**Table 1:** The count of samples in the used dataset before and after SMOTE technique

Class label	Before SMOTE	After SMOTE (oversampling)
No financial crisis	2408	2408
Financial crisis	91	2408

### Feature Selection Based on the PIO Phase

The feature selection method reduces the number of columns by selecting some sets from the original data and removing inappropriate, ineffective, and repetitive variables. Selecting features is one of the complex tasks of a large data set. It reduces the number of features by picking particular subsets of the original variables. When working with a large dataset, one of the most challenging problems is determining which features are the most important. It has been shown that covariate selection effectively removes inappropriate and redundant features. Also, it has the potential to minimize the computation time and increase the classifier performance. Feature selection using the metaheuristic algorithm has been successfully applied to financial forecastings such as Particle Swarm Optimization (PSO) and Quantum-behaved PSO.

The Pigeon Inspired Optimization algorithm normally relies on a fitness function to perform feature selection and measure the validity of solutions in a given optimization problem. The used fitness function depends on the total number of features (TF), the performance of the classification model (accuracy), the reduct length, and three corresponding weights ${w}_{1}$, ${w}_{2},$ and ${w}_{3}$ to the significance of classification performance, the sum of the three weights equals 1. Equation 9 defines the fitness function, while Eq. 10 defines the reduct weight dependent on the importance of the feature within the reduct. In this paper, we are setting ${w}_{1}=0.70$, ${w}_{2}=0.22$, and ${w}_{3}=0.08.$

9 $$Fitness\,function={w}_{1}*accuracy+{w}_{2}*{F}_{1}+{w}_{3}*\left(1-\frac{SF}{TF}\right)$$

10 $$Reduct weight={\sum }_{i}importance\left({f}_{i}\right)*\frac{ TF-SF}{TF},s.t.\,importance\left({f}_{i}\right)>threshold$$

**Fig. 3:**
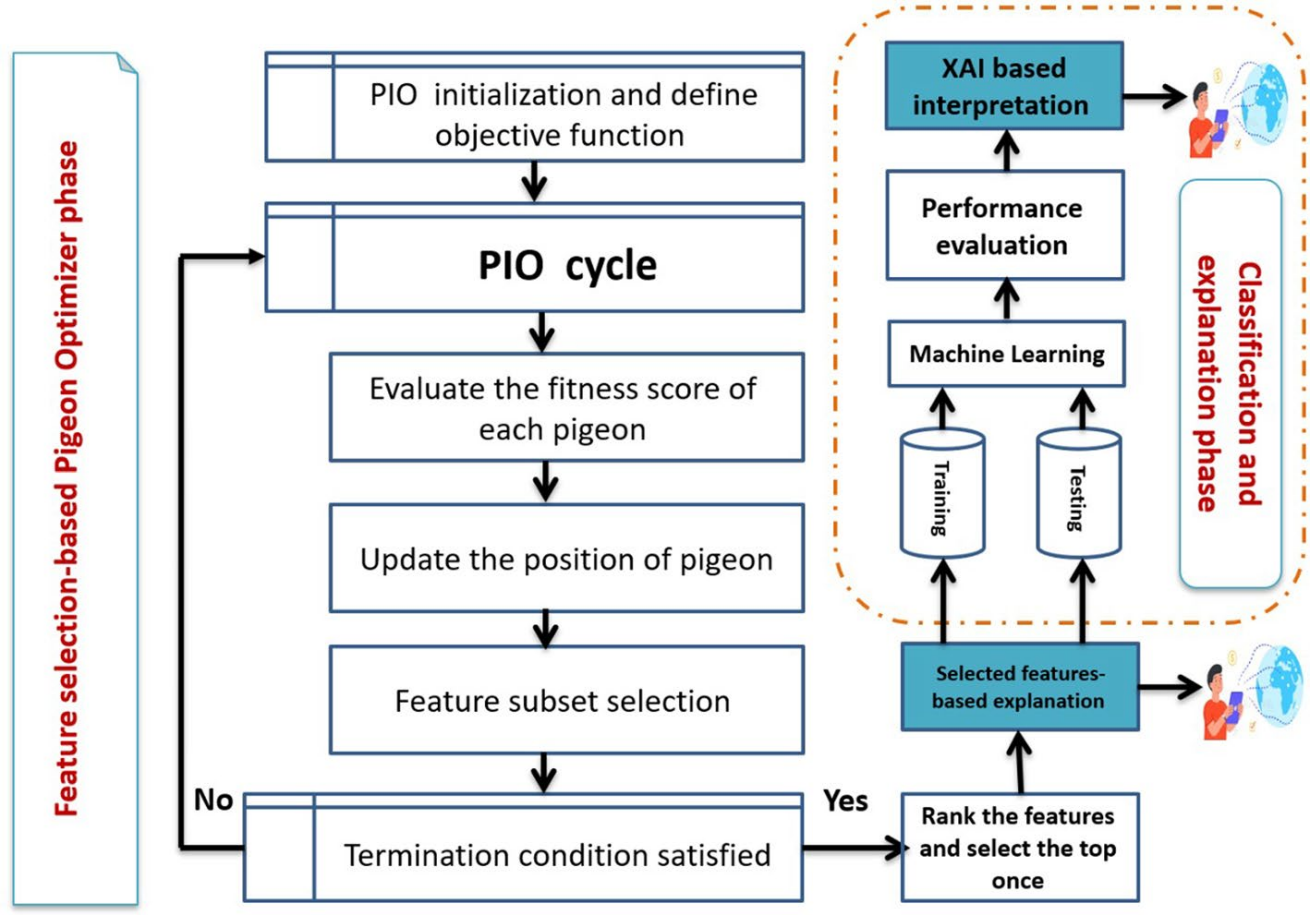
The feature selection process based on the PIO algorithm and XAI

Figure 3 illustrates the process of optimized feature selection with their explanation based on XAI interpretation. The steps for feature selection based on the PIO algorithm are: (1) Initialize parameters and define the fitness function. (2) Evaluate the fitness value of all Pigeons. (3) Each pigeon's velocity and position are continuously updated. (4) The global best position is modified with the pigeon's new position having the lowest fitness function value. (5) The stop condition is the last step in the PIO cycle. The parameters are tuned to achieve a higher level of accuracy with a lower error rate. (6) The best reducts are sorted and the top ranks are selected. (7) Classification model and XAI are used to provide interpretation for each reduct generated. (8) Finally, the proposed model produced the best reduct.

### Financial Crisis Roots Recognition Based on the Machine Learning Phase

In this phase, three different machine learning models, namely Support Vector Machine (SVM), Random Forest (RF), and Gradient Boosting (GB), are used and compared to identify the roots of the financial crisis. These models are trained without utilizing the SMOTE approach, and their performance is measured using the confusion matrix and the relative performance metrics such as precision, F1-score, and accuracy. Similarly, the three models are trained and tested after applying the SMOTE approach with tenfold cross-validation to prevent overfitting and unbalanced data problems. Finally, the SHAP approach is applied as an XAI model to explain the results of the applied machine learning algorithms in recognizing financial crisis roots.

## Experimental Results

This section evaluates the effectiveness of the proposed Explainable AI model in recognizing financial crisis roots based on the PIO algorithm using a benchmark dataset [42, 43]. The design of the proposed model is developed in Python. Whereas, testing the system's performance is carried out on a Google collab cloud equipped with a CPU running at 2.6 GHz and 32 GB of RAM. We set the number of initial values related to feature selection and optimization operations. For instance, the feature threshold is 0.1. The number of iterations in PIO is 100, and the number of pigeons in the Pigeon Swarm Algorithm equals half the number of features. The halting condition occurs when the number of iterations equals the maximum value specified previously [44, 45]**.**

The performance of the proposed model was evaluated using accuracy, precision, recall and F1-score, as outlined in Eqs. 11–14.

11 $$Accuracy=\frac{TP+TN}{TP+TN+FP+FN}$$

12 $$Precision=\frac{TP}{TP+FP}$$

13 $$Recall=\frac{TP}{TP+FN}$$

14 $$F1-Score=\frac{2\left(Precision*Recall\right)}{Precision+Recall}$$

where TP, TN, FN, and FP are truly positive, true negative, false negative, and false positive variables.

### Dataset Description

The used dataset is the JSTdatasetR3 dataset [42, 43]. This dataset was generated with considerable financial support from organizations such as the Institute for New Economic Thinking, the German Federal Ministry of Education and Research, the Volkswagen Foundation, and the European Research Council [42]. Table 2 provides a summary of this data. The meaning of attributes in the dataset is shown in Table 3. The parameters considered in the dataset collection for qualitative analysis were collected from the characteristics of industrial risk, competitiveness, credibility, financial flexibility, management risk, and operating risk.

**Table 2:** The description of the dataset

Variable	Value
Countries	['Australia', 'Belgium', 'Canada', 'Denmark', 'Italy', 'Japan', 'Germany', 'Finland', 'France', 'UK', 'Netherlands', 'Norway', 'Portugal', 'Spain', 'Switzerland', 'Sweden', 'USA']
Period	1870–2016
Rows	2499
Attributes	29
Classes	2 (0/1)

**Table 3:** The description of used features in the dataset

Features			Features		
Data attributes	Features	Meaning	Data attributes	Features	Meaning
Year	–	Year	narrowm	'F_11'	Narrow money
Country	–	Country	money	'F_12'	Broad money
iso	–	ISO 3-letter code	stir	'F_13'	Short-term interest rate
ifs	'F_0'	IFS 3-number country-code	ltrate	'F_14'	Long-term interest rate
pop	'F_1'	Population	stocks	'F_15'	Stock prices
rgdpmad	'F_2'	Real GDP per capita (PPP)	debt-GDP	'F_16'	Public debt-to-GDP ratio
rgdppc	'F_3'	Real GDP per capita	revenue	'F_17'	Government revenues
rconpc	'F_4'	Real consumption per capita	expenditure	'F_18'	Government expenditure
GDP	'F_5'	GDP	xrusd	'F_19'	USD exchange rate
iy	'F_6'	Investment-to-GDP ratio	tloans	'F_20'	Total loans to the non-financial private sector
cpi	'F_7'	Consumer prices	tmort	'F_21'	Mortgage loans to the non-financial private sector
ca	'F_8'	Current account	thh	'F_22'	Total loans to households
imports	'F_9'	Imports	tbus	'F_23'	Total loans to business
exports	'F_10'	Exports	hpnom	'F_24'	House prices

### Feature Selection Results

The used dataset has 29 features of financial crisis for 147 samples collected from 17 countries. For solving the unbalancing problem of the used dataset, SMOTE algorithm has been applied, then 80% of the samples have been used for training and 20% have been used for testing. Table 4 shows the number of samples in both of the two classes (i.e., No-Financial Crisis, and Financial Crisis) before and after applying the SMOTE approach.

**Table 4:** The count of samples in the used dataset before and after SMOTE technique

Class label	Before SMOTE	After SMOTE (oversampling)
No financial crisis	2408	2408
Financial crisis	91	2408

**Fig. 4:**
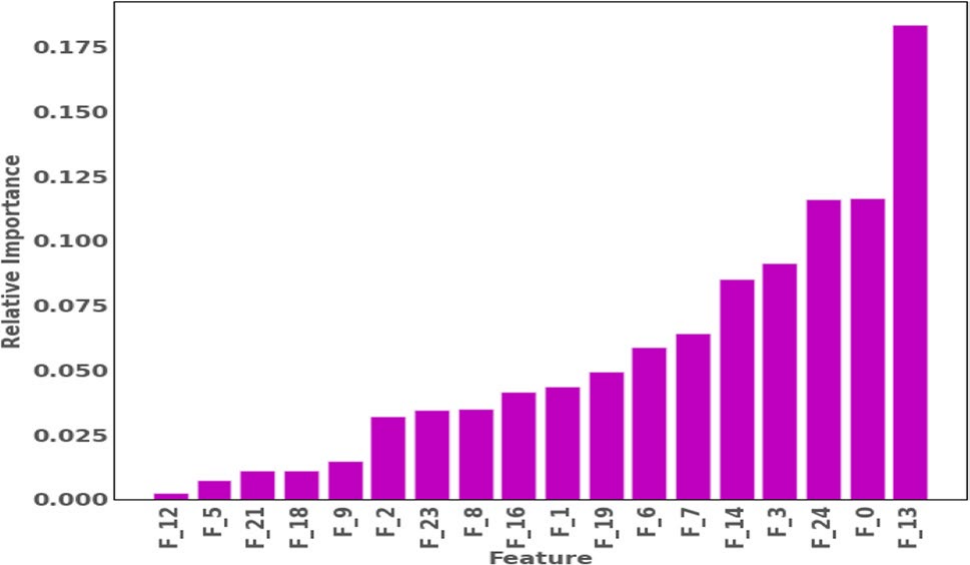
The relative importance and stability score for the reduct { F_0, F_1, F_2, F_3, F_5, F_6, F_7, F_8, F_9, F_12, F_13, F_14, F_16, F_18, F_19, F_21, F_23, and F_24.}

**Fig. 5:**
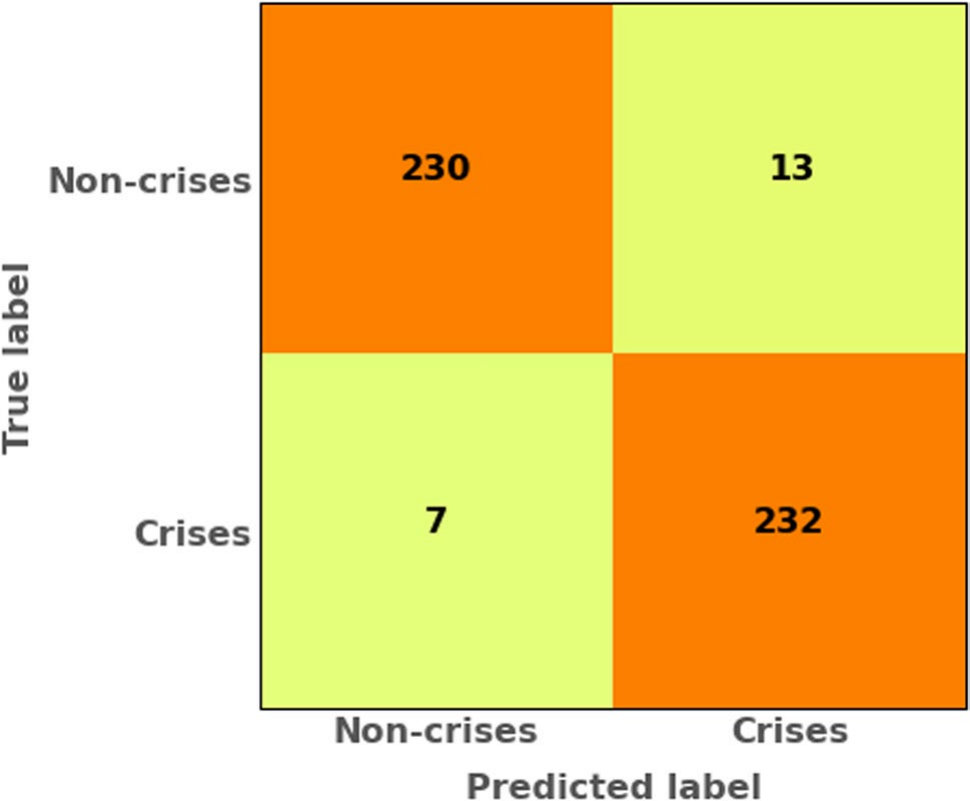
The classification results based on the reduct { F_0, F_1, F_2, F_3, F_5, F_6, F_7, F_8, F_9, F_12, F_13, F_14, F_16, F_18, F_19, F_21, F_23, and F_24.}

Table 5 presents the feature selection results of the suggested PIO method on the financial crisis dataset. Moreover, the relative importance of the most significant features is depicted in Fig. 4. F_13 has the highest importance. Moreover, the optimization results clarified that the reduct that consists of these features: { F_0, F_1, F_2, F_3, F_5, F_6, F_7, F_8, F_9, F_12, F_13, F_14, F_16, F_18, F_19, F_21, F_23, and F_24} has a high stability value of 0.936. This means that they are the most significant features that can accurately recognize financial crises. Moreover, the classification results based on the built-in Gradient Boosting classifier in the PIO algorithm using the mentioned reduct of features are depicted in the confusion matrix in Fig. 5. Table 6 summarizes training and testing results of the performance of the proposed PIO model as well as the weight of the selected reduct selected from the financial crisis dataset, which is the most effective in recognizing financial crisis roots.

**Table 5:** Feature selection results based on PIO optimization methodology

Fn value	Xgb (Global)					Xpg(local)				
Fitness value	Accuracy	Precision	Recall	F1	Reduct	Accuracy	Precision	Recall	F1	Reduct
0.951463	0.982	0.978	0.986	0.982	[0, 3, 7, 8, 9, 11, 16, 19, 23, 24]	0.981	0.972	0.989	0.981	[0, 1, 7, 9, 13, 14, 18, 20, 21, 23]
0.951463	0.982	0.978	0.986	0.982	[0, 3, 7, 8, 9, 11, 16, 19, 23, 24]	0.970	0.965	0.975	0.970	[2, 5, 7, 13, 15, 16, 18]
0.951463	0.982	0.978	0.986	0.982	[0, 3, 7, 8, 9, 11, 16, 19, 23, 24]	0.983	0.977	0.989	0.983	[0, 1, 4, 6, 9, 13, 15, 17, 18, 20, 23]
0.951463	0.982	0.978	0.986	0.982	[0, 3, 7, 8, 9, 11, 16, 19, 23, 24]	0.989	0.985	0.993	0.989	[0, 3, 4, 7, 9, 12, 13, 14, 18, 19, 20, 24]
0.958672	0.990	0.985	0.994	0.990	[0, 1, 2, 6, 13, 14, 15, 19, 23, 24]	0.990	0.985	0.994	0.990	[0, 1, 2, 6, 13, 14, 15, 19, 23, 24]
0.958672	0.990	0.985	0.994	0.990	[0, 1, 2, 6, 13, 14, 15, 19, 23, 24]	0.986	0.979	0.994	0.986	[0, 1, 3, 4, 7, 8, 9, 11, 13, 15, 24]
0.958672	0.990	0.985	0.994	0.990	[0, 1, 2, 6, 13, 14, 15, 19, 23, 24]	0.986	0.977	0.996	0.986	[0, 4, 5, 6, 9, 13, 14, 16, 17, 18, 19, 21, 23]
0.958672	0.990	0.985	0.994	0.990	[0, 1, 2, 6, 13, 14, 15, 19, 23, 24]	0.979	0.967	0.991	0.979	[1, 2, 4, 8, 10, 13, 17, 18, 21]
0.958672	0.990	0.985	0.994	0.990	[0, 1, 2, 6, 13, 14, 15, 19, 23, 24]	0.972	0.962	0.982	0.972	[0, 5, 10, 13, 14, 20, 22]
0.958672	0.993	0.993	0.993	0.993	[0, 1, 2, 3, 5, 6, 7, 8, 9, 12, 13, 14, 16, 18, 19, 21, 23, 24]	0.993	0.993	0.993	0.993	[0, 1, 2, 3, 5, 6, 7, 8, 9, 12, 13, 14, 16, 18, 19, 21, 23, 24]

**Table 6:** Training and testing results of the proposed model based on the optimized reduct of features: [0, 1, 2, 3, 5, 6, 7, 8, 9, 12, 13, 14, 16, 18, 19, 21, 23, 24]

Train				Test						
Accuracy	Precision	Recall	F1	Accuracy	Precision	Recall	F1	Reduct	Weight	Reduct size
0.993	0.993	0.993	0.993	0.959	0.947	0.971	0.959	[0, 1, 2, 3, 5, 6, 7, 8, 9, 12, 13, 14, 16, 18, 19, 21, 23, 24]	0.936	18

**Fig. 6:**
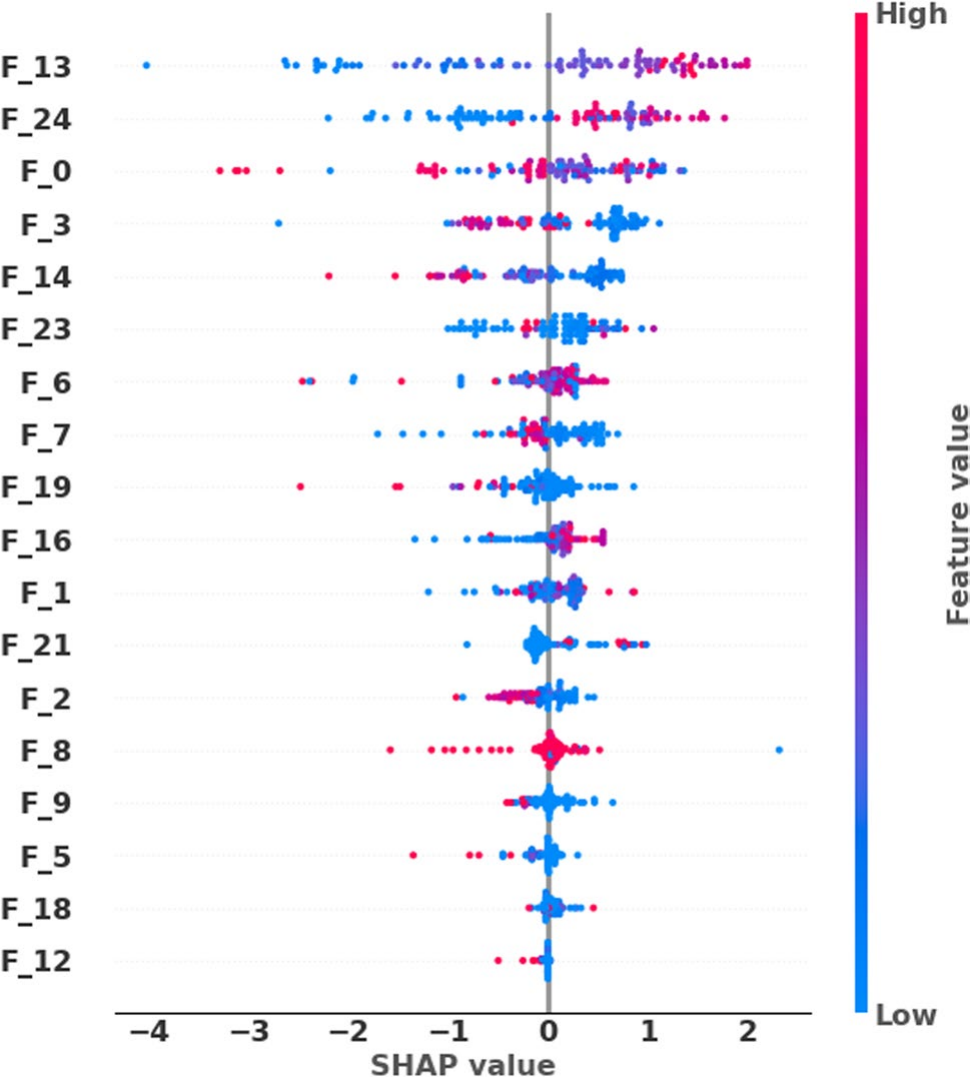
XAI results using SHAP functions to the optimal reduct of features have the most influence on financial crisis prediction results

For interpreting and understanding the impact of each feature in the set of optimal features [0, 1, 2, 3, 5, 6, 7, 8, 9, 12, 13, 14, 16, 18, 19, 21, 23, 24] on the financial crisis prediction results, an Explainable AI (XAI) technique called SHAP is used to plot the interpretations of those features. SHAP is a powerful mathematical tool for interpreting the output of machine learning algorithms. It is based on the principles of game theory and allows for the estimation of the contribution of each feature to the prediction results generated by a model. SHAP is a valuable tool for understanding how a model is making its predictions and can be used to explain the output of any machine learning algorithm. SHAP can identify the most important features and their influence on the model prediction. Figure 6 depicts the XAI results using the SHAP function. The horizontal axis indicates the feature's influence on prediction (low or high). The color represents the feature's significance (red means high significance, and blue low significance). It is clear from Fig. 6 that feature F13 (i.e., short-term interest rate) has the most influence on financial crisis prediction results.

### Crisis Roots Recognition and Classification Results

The availability of consistent financial data that has a sufficient history to allow for standard analysis is frequently lacking. Therefore, monitoring the financial risk that is present in the global economy seems to be a difficult challenge. The "shadow banking" industry is growing, and technological firms are also offering payment and credit services, which means that both the source of risk and the rules governing finance and financial reporting are rapidly evolving.

Forecasting financial crises are important for financial firms, which attempt to reduce possible losses by estimating prospective risks and avoiding new credit applications when the default risk exceeds a predetermined acceptance level. Forecasting financial crises can help prevent potential losses. This method is also known as a "credit default classification procedure" because it determines whether a consumer is "non-default" or "default" when he pays off the loan. In other words, it classifies the customer's credit status. The precision of the model that has been proposed is necessary to evaluate the productiveness and profitability of a financial company.

In this work, we address the challenge of evaluating the general healthiness of financial institutions, thereby contributing to the identification of the roots of the financial crisis. The model trained and performed with the PIO Optimization Algorithm feature selection had been successful, with the objectives of minimization of features and maximization of performance. The suggested PIO method has two stages: the first is the selection of the best reduct, and the second is the classification of financial crisis data.

**Fig. 7:**
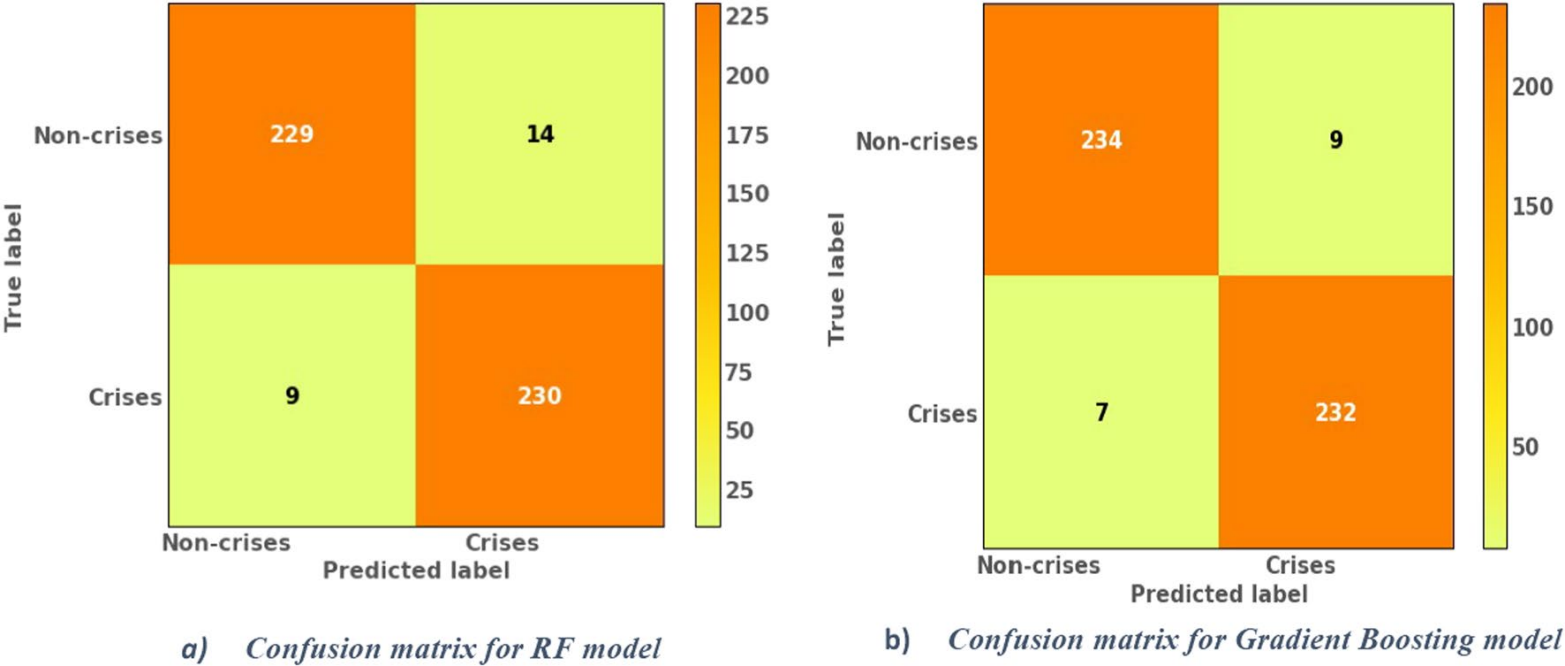
Confusion matrices of classification results: a) Confusion matrix for Random Forest model, b) confusion matrix for Gradient Boosting model

After obtaining the optimal reduct of features that have the most influence on financial crisis prediction, the Gradient Boosting classifier is compared with the random forest (RF) classifier in classifying and recognizing financial crisis roots to validate its efficiency. Table 7 summarizes the comparison results of the performance metrics of Gradient Boosting, and Random Forest (RF), on the dataset after applying the feature selection step. The Gradient Boosting model achieved better performance in recognizing financial crisis roots than Random Forest. This result is confirmed through the confusion matrix results of both of the two applied classifiers, the Gradient Boosting, and Random Forest as depicted in Fig. 7.

**Table 7:** Comparative performance results of Gradient Boosting and Random Forest classifiers

Classifier	Classification results				
	Reduct	Accuracy	Precision	Recall	F1-Score
PIO + XAI model	[0, 1, 2, 3, 5, 6, 7, 8, 9, 12, 13, 14, 16, 18, 19, 21, 23, 24]	0.959	0.947	0.971	0.959
Gradient Boosting		0.967	0.962	0.971	0.967
Random Forest		0.952	0.942	0.962	0.952

## Discussion

Although many works have been proposed to solve financial crisis prediction issues [1, 15–33], very little work has used the concept of *Explainable Artificial Intelligence (XAI)* in predicting financial crises. The first question in this study sought to determine the most important features which have a big influence in predicting a financial crisis. To answer this question, the Pigeon Swarm Optimizer (PIO) algorithm has been applied to the used dataset [42] to optimize the feature selection operation. The optimization results clarified that the highlighted features in Table 8 { F_0, F_1, F_2, F_3, F_5, F_6, F_7, F_8, F_9, F_12, F_13, F_14, F_16, F_18, F_19, F_21, F_23, and F_24} are the most significant features that can accurately recognize financial crisis roots according to ten rounds of optimization using ten fitness values.

**Table 8:** The optimized features used to recognize financial crisis roots

Features			Features		
Data attributes	Feature ID	Meaning	Data attributes	Feature ID	Meaning
Year	–	Year	narrowm	'F_11'	Narrow money
Country	–	Country	money	'F_12'	Broad money
iso	–	ISO 3-letter code	stir	'F_13'	Short-term interest rate
ifs	'F_0'	IFS 3-number country-code	ltrate	'F_14'	Long-term interest rate
pop	'F_1'	Population	stocks	'F_15'	Stock prices
rgdpmad	'F_2'	Real GDP per capita (PPP)	debt-GDP	'F_16'	Public debt-to-GDP ratio
rgdppc	'F_3'	Real GDP per capita	revenue	'F_17'	Government revenues
rconpc	'F_4'	Real consumption per capita	expenditure	'F_18'	Government expenditure
GDP	'F_5'	GDP	xrusd	'F_19'	USD exchange rate
iy	'F_6'	Investment-to-GDP ratio	tloans	'F_20'	Total loans to the non-financial private sector
cpi	'F_7'	Consumer prices	tmort	'F_21'	Mortgage loans to the non-financial private sector
ca	'F_8'	Current account	thh	'F_22'	Total loans to households
Imports	'F_9'	Imports	tbus	'F_23'	Total loans to business
Exports	'F_10'	Exports	hpnom	'F_24'	House prices

Moreover, the optimization results clarified that the *short-term interest rates feature* (i.e., F_13) is the most important feature by which financial crisis roots can be detected (as depicted previously in Fig. 4). Another important finding was that the classification results based on the built-in *Gradient Boosting classifier* in the PIO algorithm achieved training and testing accuracy of 99% and 96.7%, respectively, in recognizing financial crisis roots using the reduct of features: { F_0, F_1, F_2, F_3, F_5, F_6, F_7, F_8, F_9, F_12, F_13, F_14, F_16, F_18, F_19, F_21, F_23, and F_24}.

On the question of interpreting the features influence in classification results, Fig. 6 presented the influence interpretations of the optimized features on financial crisis classification. The used XAI model showed that the short-term interest rates feature (i.e., F_13) is the most important feature by which financial crisis roots can be detected (as depicted previously in Fig. 6).

**Fig. 8:**
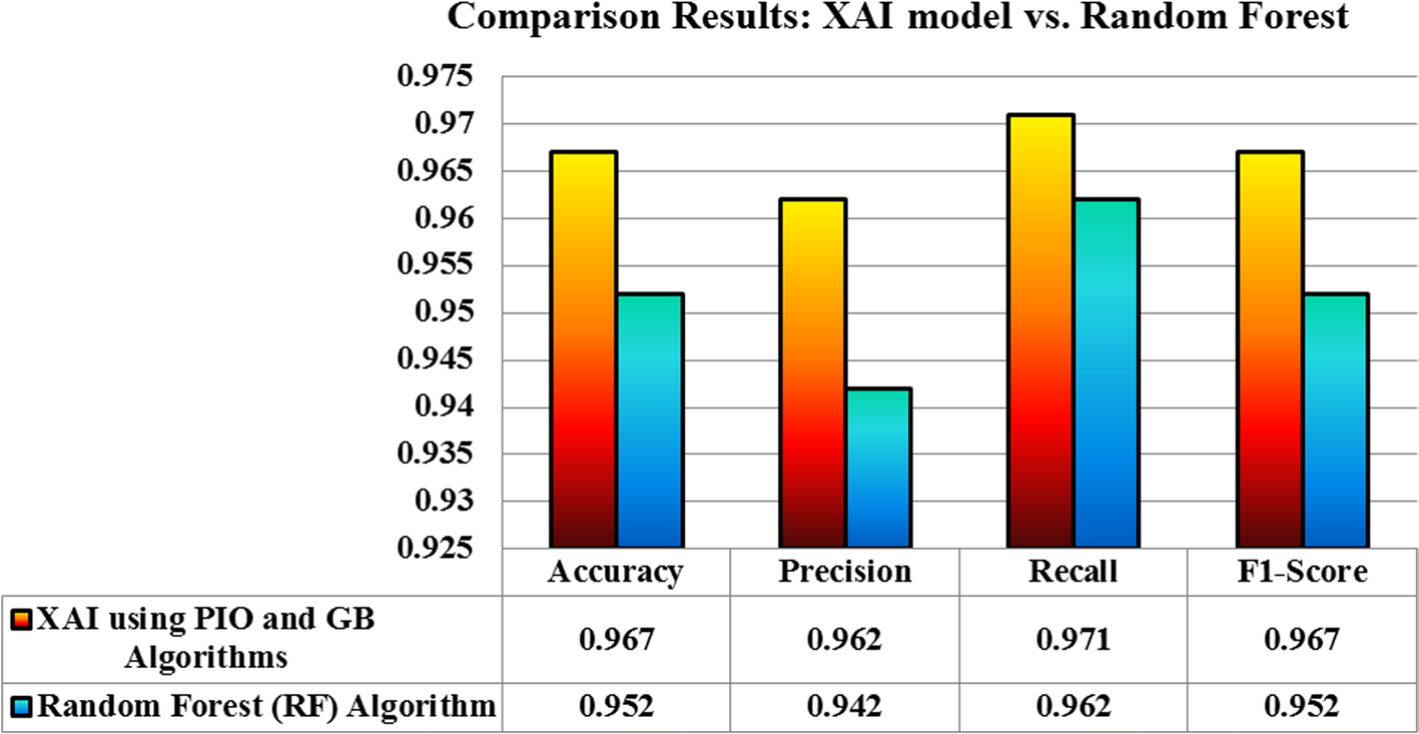
Comparison results: the proposed XAI model vs. Random Forest classifier in recognizing financial crisis roots

To validate the efficiency of the proposed XAI model in recognizing financial crisis roots, the Random Forest (RF) classifier has been applied using the same reduct of optimized features. The obtained results confirmed the superiority of the proposed XAI model in recognizing financial crisis roots based on PIO, and Gradient Boosting algorithms as depicted in Fig. 8.

Table 9 summarizes the comparison results between the state-of-art models and the proposed XAI model. The classification performances metrics ( e.g., accuracy, precision, recall, and F1-score) clarify that the proposed XAI model has the best performance in recognizing and interpreting financial crisis classification results compared with the state-of-art models, NN [46], LASSO-CART [47], and Decision Trees [48], Extreme Gradient Boosting [49] models.

**Table 9:** Comparison between the state-of-art models and the proposed XAI model

Classifier	Classification results			
	Accuracy	Precision	Recall	F1-score
Neural Networks (NN) [46]	0.91	0.92	0.91	0.91
LASSO-CART [47]	89.74	NA	NA	NA
Decision Trees model [48]	0.9524	0.9355	0.9667	NA
Extreme Gradient Boosting [49]	0.9566	0.8726	0.8354	0.8536
**Gradient Boosting**	**0.967**	**0.962**	**0.971**	**0.967**
**The proposed XAI model**	**0.959**	**0.947**	**0.971**	**0.959**

Bold values indicate better results than other classifiers

## Conclusion

The purpose of the current study was to investigate the efficiency of a proposed XAI model to recognize financial crisis roots based on Pigeon Optimization and Gradient Boosting classifier. The investigation of feature selection optimization has shown that the *short-term interest rates feature* is the most important feature by which financial crisis roots can be detected. On the other hand, the following features ids: [0, 1, 2, 3, 5, 6, 7, 8, 9, 12, 13, 14, 16, 18, 19, 21, 23, and 24] are the most reduct by which financial crisis roots can be detected. Another important finding was the high value of training and testing accuracy in recognizing financial crisis roots. The classification results showed that the built-in Gradient Boosting classifier in the PIO algorithm achieved training and testing accuracy of 99% and 96.7%, respectively, in recognizing financial crisis roots, which is an efficient and better performance compared to the Random Forest classifier. A further study with more focus on other features that can influence financial crisis roots is needed. In addition, other optimizer techniques can be used to perform better efficient feature selection optimization than the PIO algorithm. Moreover, deep learning algorithms using various designs of neural networks can be used to enhance the classification performance in recognizing financial crisis roots. In addition, predicting banks' bankruptcy is also an important challenge for future research.

## Data Availability

The supporting data are the JSTdatasetR3 dataset which is available in the ref [36].
